# Evaluation of a Quantitative Real-Time PCR Assay to Measure HIV-Specific Mucosal CD8+ T Cell Responses in the Cervix

**DOI:** 10.1371/journal.pone.0013077

**Published:** 2010-10-07

**Authors:** Duncan Chege, Yijie Chai, Sanja Huibner, Lyle McKinnon, Charles Wachihi, Makubo Kimani, Walter Jaoko, Joshua Kimani, T. Blake Ball, Francis A. Plummer, Rupert Kaul, Anuradha Rebbapragada

**Affiliations:** 1 Department of Medicine, University of Toronto, Toronto, Ontario, Canada; 2 Departments of Medicine, University Health Network, Toronto, Ontario, Canada; 3 Public Health Laboratory-Toronto, Ontario Agency for Health Protection and Promotion, Toronto, Ontario, Canada; 4 Department of Laboratory Medicine and Pathobiology, University of Toronto, Toronto, Ontario, Canada; 5 Department of Medical Microbiology, University of Manitoba, Winnipeg, Canada; 6 Laboratory for HIV Immunology, National HIV and Retrovirology Laboratories, Public Health Agency of Canada, Winnipeg, Canada; 7 Department of Medical Microbiology, University of Nairobi, Nairobi, Kenya; New York University, United States of America

## Abstract

Several candidate HIV vaccines aim to induce virus-specific cellular immunity particularly in the genital tract, typically the initial site of HIV acquisition. However, standardized and sensitive methods for evaluating HIV-specific immune responses at the genital level are lacking. Therefore we evaluated real-time quantitative PCR (qPCR) as a potential platform to measure these responses. β-Actin and GAPDH were identified as the most stable housekeeping reference genes in peripheral blood mononuclear cells (PBMCs) and cervical mononuclear cells (CMCs) respectively and were used for normalizing transcript mRNA expression. HIV-specific cellular T cell immune responses to a pool of optimized CD8+ HIV epitopes (HIV epitope pool) and Staphylococcal enterotoxin B (SEB) superantigen control were assayed in HIV infected PBMC by qPCR, with parallel assessment of cytokine protein production. Peak HIV-specific mRNA expression of IFNγ, IL-2 and TNFα occurred after 3, 5 and 12 hours respectively. PBMCs were titrated to cervical appropriate cell numbers to determine minimum required assay input cell numbers; qPCR retained sensitivity with input of at least 2.5×10^4^ PBMCs. This optimized qPCR assay was then used to assess HIV-specific cellular T cell responses in cytobrush-derived cervical T cells from HIV positive individuals. SEB induced IFNγ mRNA transcription was detected in CMCs and correlated positively with IFNγ protein production. However, qPCR was unable to detect HIV-induced cytokine mRNA production in the cervix of HIV-infected women despite robust detection of gene induction in PBMCs. In conclusion, although qPCR can be used to measure *ex vivo* cellular immune responses to HIV in blood, HIV-specific responses in the cervix may fall below the threshold of qPCR detection. Nonetheless, this platform may have a potential role in measuring mitogen-induced immune responses in the genital tract.

## Introduction

Globally, 33 million people are now infected with the Human Immunodeficiency Virus (HIV) [1] and more than 90% of new infections occur across the mucosal lining of the genital or gastrointestinal tract [2]. Women now account for most new HIV infections [2], [3], [4], [5], with acquisition usually occurring across the cervico-vaginal mucosa during sex [2]. HIV-specific T cell responses are an important component of the host immune response against HIV, and have been described in the genital mucosa of both HIV-infected and exposed but uninfected women [6], [7], [8], [9], [10]. However, the role of genital HIV-specific cellular immunity in HIV susceptibility and transmission remains poorly defined, in part, due to methodological limitations [5], [11]. Currently, several candidate HIV vaccines aim to induce virus-specific cellular immune cytotoxic T lymphocyte (CTL) protection [12], [13], [14], [15]. Therefore, the ability to monitor HIV-specific cellular immune responses in the female genital tract will be important in monitoring and evaluating these HIV vaccines, and may help to characterize the mucosal pathogenesis of HIV transmission and susceptibility. Unfortunately, conventional cellular immune assay platforms lack the sensitivity to measure these mucosal responses in a reliable and reproducible way [16]. This is due in large part to the low cell numbers obtained from standard sampling techniques such as the cervical cytobrush or cervico-vaginal lavage [6], [8], [9], [10]. *In vitro* expansion of cervical cells to overcome these limitations is possible, despite the issue of potential fungal/bacterial contamination when cervical lymphocytes are maintained in long-term culture [9]. However, cervical cell expansion precludes the assessment of *ex vivo* mucosal immune response frequency, and the preferential expansion of certain memory subsets using this technique means that the repertoire and memory phenotype of cervical cells obtained via expansion may not reflect *in vivo* mucosal T cell populations [11], [17]. In addition, assay platforms such as ELISPOT, chromium (Cr_51_) lysis and proliferation (CFSE dilution or H_3_-thymidine uptake) have a limited capacity to measure multiple immune functions. More recently, multi-parameter flow cytometry assays in peripheral blood mononuclear cells (PBMC) from HIV infected patients have demonstrated that the breadth and quality of immune responses appear more important than the quantity of responses in HIV control [18]. Therefore relying on measurements of narrowly defined HIV-specific immune responses might overlook the spectrum of responses needed for efficient HIV control. However, the recovery of few cervical mononuclear cells (CMCs) may still challenge the feasibility of flow cytometry in measuring HIV-specific genital tract immune responses [8].

Quantitative Real-Time PCR (qPCR) is a molecular technique that might be adapted to overcome several of these obstacles. The technique is highly sensitive [19], [20], and in theory could simultaneously assay the induction of multiple immune genes in response to antigenic stimulation despite low sample cellular input. qPCR has previously been utilized to measure basal cytokine mRNA transcript levels in unstimulated cervical cells obtained by cytobrush sampling [21], [22], as well as in clonally expanded PBMCs [20]. In addition, qPCR has frequently been used to assay systemic antigen-specific immune responses in blood [19], [20], [23]. However, there are no published reports using this platform to evaluate genital mucosal immune responses. We therefore adapted the qPCR assay to define the optimal kinetics for HIV-specific mRNA gene induction, evaluate qPCRs suitability to measure these immune responses using cervical-appropriate reduced cell numbers in a PBMC model, and compared our gene expression results with conventional protein measurement assays. This optimized qPCR assay was then used to assess HIV-specific cellular T cell responses in cervical cytobrush-derived T cells from HIV positive individuals.

## Results

### Selection of housekeeping genes

Reliable comparisons of gene expression between samples require the normalization of input amounts, reverse transcription and amplification reaction efficiencies. PBMCs and CMCs from 5 HIV infected individuals were incubated for 6 hours in R10 medium alone, HIV epitope pool, or SEB, and mRNA was evaluated for the expression of the commonly-used housekeeping genes including β-actin, B2M, GAPDH, HPRT and TBP [24], [25]. Housekeeping gene expression stability across different tissues and treatments was evaluated using the NormFinder analysis algorithm which identified β -actin as the most stable HK gene in PBMCs (stability value, 1622.17), while GAPDH was the most stable in CMCs (stability value, 104.92). In PBMCs, the top 4 housekeeping genes appeared to have a narrower spread in stability values (range, 254.6 stability units) suggesting their stability their expression remains constant in this cell type. However in CMCs, GAPDH housekeeping gene emerged as the most stable gene with no closely comparable housekeeping gene among the top 4 evaluated genes (range, 433.8 stability units). This finding emphasizes the need to validate candidate housekeeping genes in each sample source of interests. In both cell types, B2M gene was the least stable. Table 1 summarises the ranking of HK genes from the most to least stable. GAPDH and β-actin genes were selected as our respective reference HK gene for normalization of cytokine gene induction in CMC and PBMC cell types respectively.

**Table 1:** NormFinder rankings of candidate reference housekeeping genes.

Rank	CMC	Stability value	PBMC	Stability value
**1**	GAPDH	104.9	β-Actin	1622.2
**2**	β-Actin	538.7	HPRT	1833.8
**3**	HPRT	574.6	TBP	1859.2
**4**	TBP	588.1	GAPDH	1876.8
**5**	B2M	3563.8	B2M	6997.3

β-2-Microglobulin (B2M); TATA box binding protein (TBP); Glyceraldehyde-3-phosphate dehydrogenase (GAPDH); human hypoxanthine phosphoribosyltransferase (HPRT).

Genes are ranked from the most stable (1) to the least stable (5) according to Norm-Finder gene-stability analysis algorithms. Housekeeping gene stability was evaluated by comparing between 5 different HIV+ PBMC or CMC samples and within an individual treated with either Negative control (R10 media,) HIV antigen (HIV epitope pool) or positive control (SEB).

### Time course of HIV-specific mRNA induction and cytokine release

To characterize virus-specific cellular T cell cytokine induction kinetics, one million PBMCs from 4 HIV positive individuals were stimulated with the predefined HIV CD8+ peptide epitope pool for 12 hours; and *ex vivo* cultured cells were harvested at hourly intervals for the first six hours and then once every two hours up to 12 hours. At this point the experiment was terminated since our goal was to mimic the time course of cervical T cell assays, and in our experience these may become contaminated in the context of more prolonged incubation. mRNA and protein levels of IFNγ, TNFα and IL-2 were quantified in parallel. Cytokine mRNA and protein kinetics were defined in PBMCs because of abundant cell yields in this compartment.

**Figure 1:**
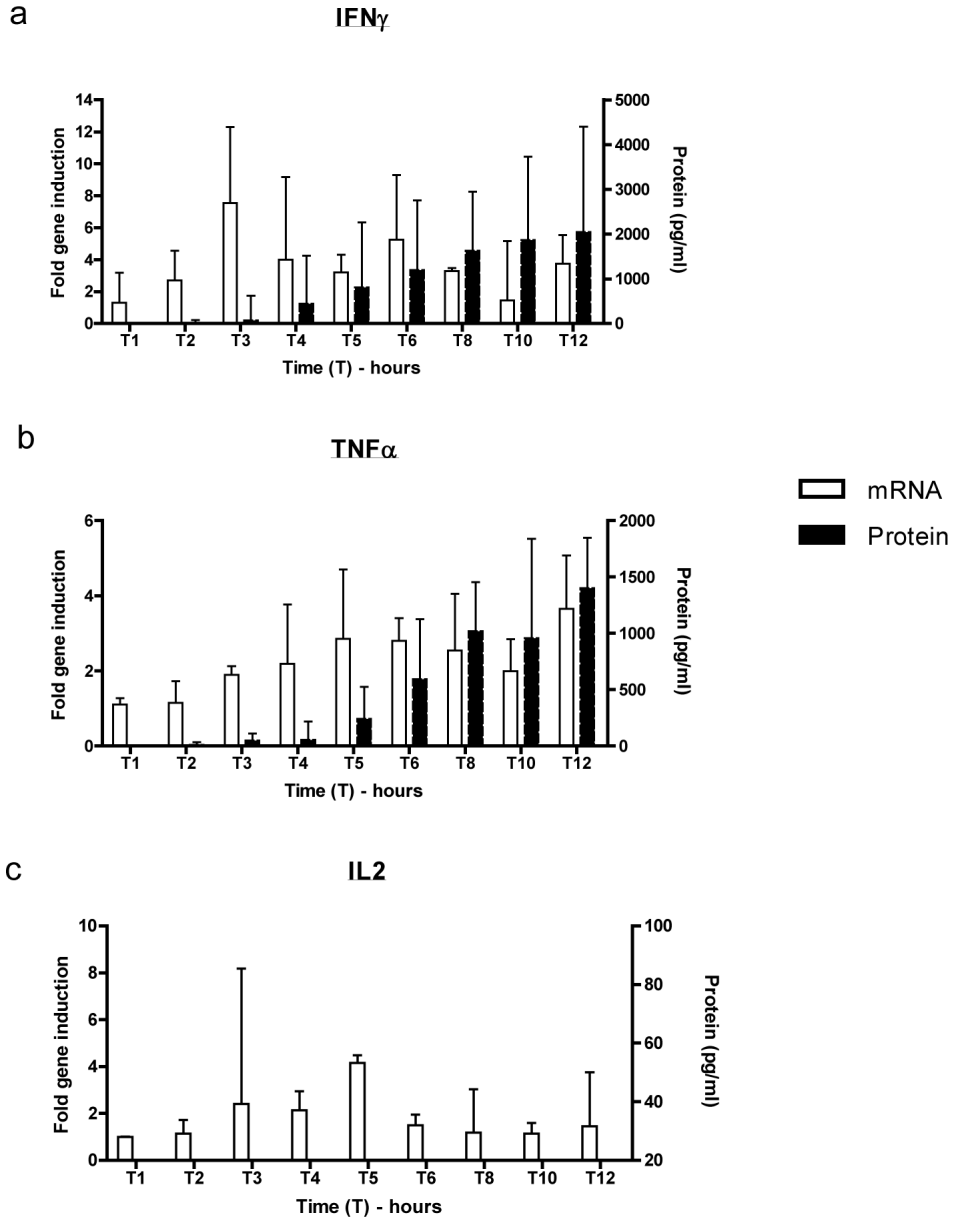
Comparison of the cytokine kinetics of HIV-specific mRNA and protein induction. 1×10^6^ peripheral blood mononuclear cell samples from 4 HIV infected individuals were incubated with HIV CD8+ T cell HIV optimized epitope antigen e (HIV epitope pool) and mRNA and protein induction assayed via qPCR and CBA respectively. Bars represent median values with lines showing interquartile ranges measuring HIV-specific IFNγ (a), TNFα (b) and IL-2 (c) mRNA and protein induction kinetics are shown. Background levels of protein have been subtracted from reported values above.

IFNγ was the most abundantly induced cytokine mRNA in all PBMC stimulation conditions, followed by TNFα and IL-2 respectively. Median HIV-specific mRNA induction profiles differed for each cytokine (Figure 1): IFNγ peaked after 3 hours of stimulation (fold induction 7.8) and IL-2 peaked after 5 hours (fold induction 3.9). TNFα displayed a bimodal induction profile with peaks, with the first mRNA induction peak occurring after 5–6 hours (fold induction 3.0–3.3 fold) and the second after 12 hours (fold induction 3.7). In contrast, supernatant protein levels of both IFNγ and TNFα increased steadily throughout the 12-hour experimental period. No IL-2 protein was detected in cell supernatants despite early mRNA induction.

Based on the above optimal cytokine kinetics, we selected to incubate PBMCs and CMCs with HIV epitope pool for 6 hours. This secondary optimal time point would allow for maximal measurement of all cytokine gene induction and protein production simultaneously.

### Input cell numbers and measurement of cellular T cell responses by qPCR

**Figure 2:**
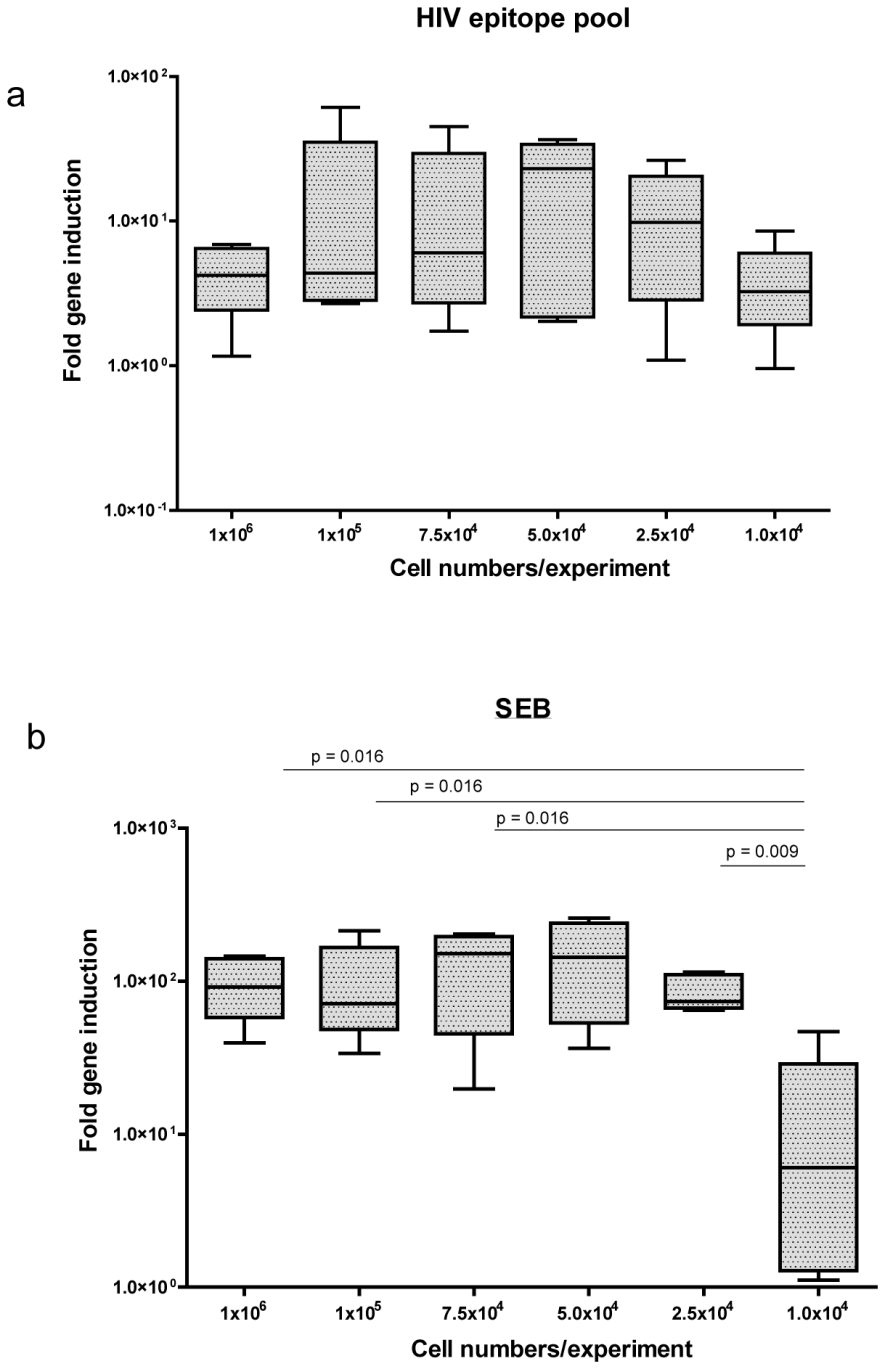
Effect of blood mononuclear cell input number on qPCR assay. A box and whiskers plot showing peripheral blood mononuclear cell samples from 5 HIV infected subjects which were plated at varying concentrations ranging from 1×10^6^ to 1×10^4^ cells/experiment and treated with R10 media, HIV epitope pool antigen or SEB positive control for 6 hours and IFNγ mRNA fold expression assayed using qPCR. Horizontal lines represent median values. (a) HIV-specific response did not significantly decrease when cell numbers decreased from 1×10^6^ to 1×10^4^ cells/experiment. (b) However, the magnitude of SEB induced IFNγ mRNA induction significantly decreased when less than 2.5×10^4^ cells/experiment were used.

We next examined the ability of the qPCR assay platform to detect HIV-specific cellular T cell responses in the context of limited input cell numbers. Five chronically HIV-infected, antiretroviral therapy-naïve participants with >350 CD4+ T cell counts/mm^3^ were randomly selected for these assays. All participants demonstrated robust HIV-specific immune responses with an input cell number of 1×10^6^ PBMC/well. PBMCs were plated at various concentrations (1×10^6^, 1×10^5^, 7.5×10^4^, 5.0×10^4^, 2.5×10^4^ and 1×10^4^ input cells/experiment) to model expected ranges of cervical cell yields from cytobrush sampling, incubated for 6 hours with R10 medium alone, SEB or the predefined HIV epitope pool, and IFNγ mRNA induction was quantified by qPCR. Robust detection of HIV-specific IFNγ mRNA was seen across the full range of input cell numbers (Figure 2a), with median HIV-specific mRNA induction levels ranging from 4.2 fold (at 1×10^6^ input PBMCs) to 3.2 fold (with 1×10^4^ input PBMCs). Likewise, SEB-induced IFNγ mRNA induction levels remained constant across higher input cell numbers, from 91.6 fold (at 1×10^6^ input PBMCs) to 73.7 fold (at 2.5×10^4^ input PBMCs). However, SEB-induced IFNγ induction dropped off rapidly when less than 2.5×10^4^ input cells/experiment were utilized, to just 6.0 fold above background (Figure 2b), but was still higher than HIV antigen specific induction levels at the same cell concentration. These findings suggest that even at low numbers of input cells, modeled after cervical cytobrush cell yields, qPCR is capable of detecting both antigen and superantigen induced cytokine mRNA.

### Evaluation of HIV-specific cervical cell immune responses using qPCR

Having established optimal cytokine kinetics and cell number sensitivity in PBMC, we next evaluated the ability of the qPCR platform to measure *ex vivo* cervical HIV-specific immune responses. Cervical cytobrushes and PBMCs were collected from 13 HIV-infected women in whom robust HIV-specific IFNγ T cell responses had been detected in blood with an input number of 1×10^6^ PBMC/well. Cytokine gene induction was assayed by qPCR after 6 hours of incubation with media alone, SEB or HIV epitope pool, and cytokine protein levels in supernatant were assayed in parallel by M-ELISA.

**Figure 3:**
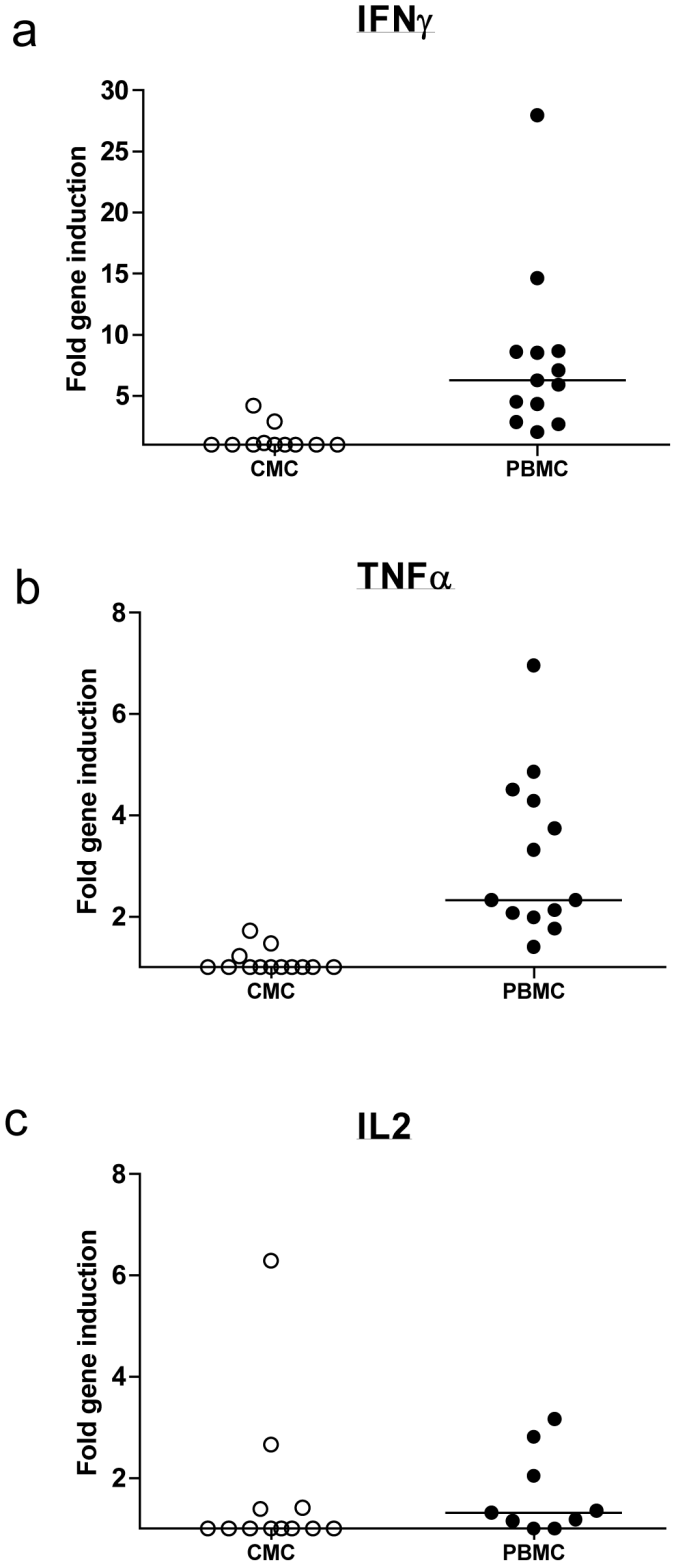
HIV-specific cytokine gene induction in cervical and blood mononuclear cells from HIV infected participants. (a) IFNγ, (b) TNFα and (c) IL-2 mRNA induction in cervical mononuclear cells (CMCs) and peripheral blood mononuclear cells (PBMCs) from 13 HIV infected women following incubation with HIV pool. Horizontal lines represent median values. The qPCR assay readily detected HIV-specific cytokine immune responses in PBMCs but was unable to do so in CMCs.

**Figure 4:**
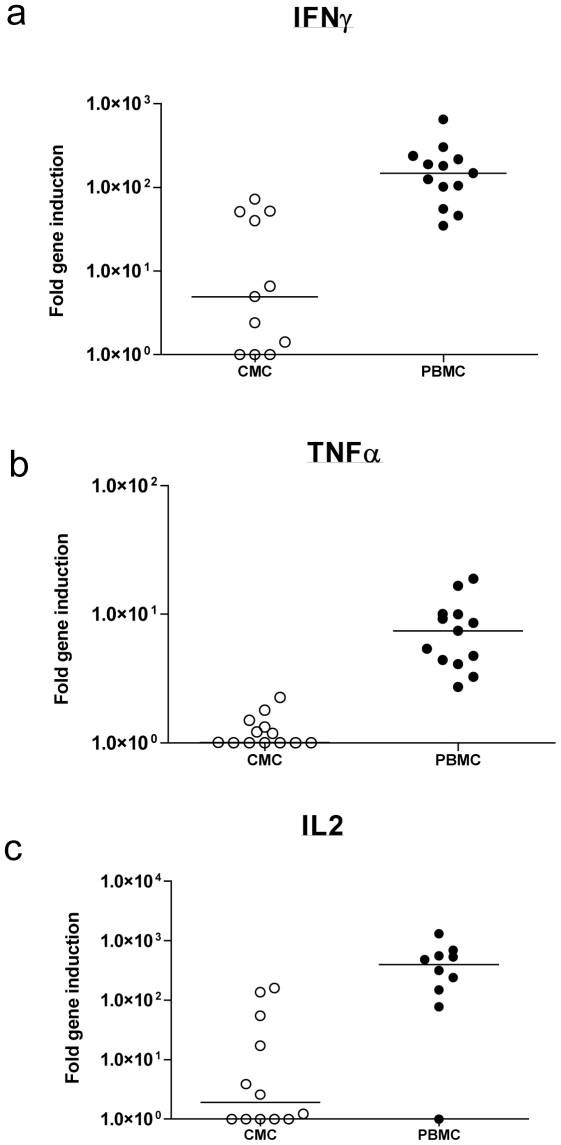
SEB superantigen mRNA cytokine induction in cervical and blood mononuclear cells from HIV infected participants. (a) IFNγ, (b) TNFα and (c) IL-2 mRNA induction in cervical mononuclear cells (CMCs) and peripheral blood mononuclear cells (PBMCs) from 13 HIV infected women following incubation with superantigen. Horizontal lines represent median values. The qPCR assay detected IFNγ and IL-2 mRNA in CMCs but not TNFα, while all cytokines were detected in PBMCs.

**Figure 5:**
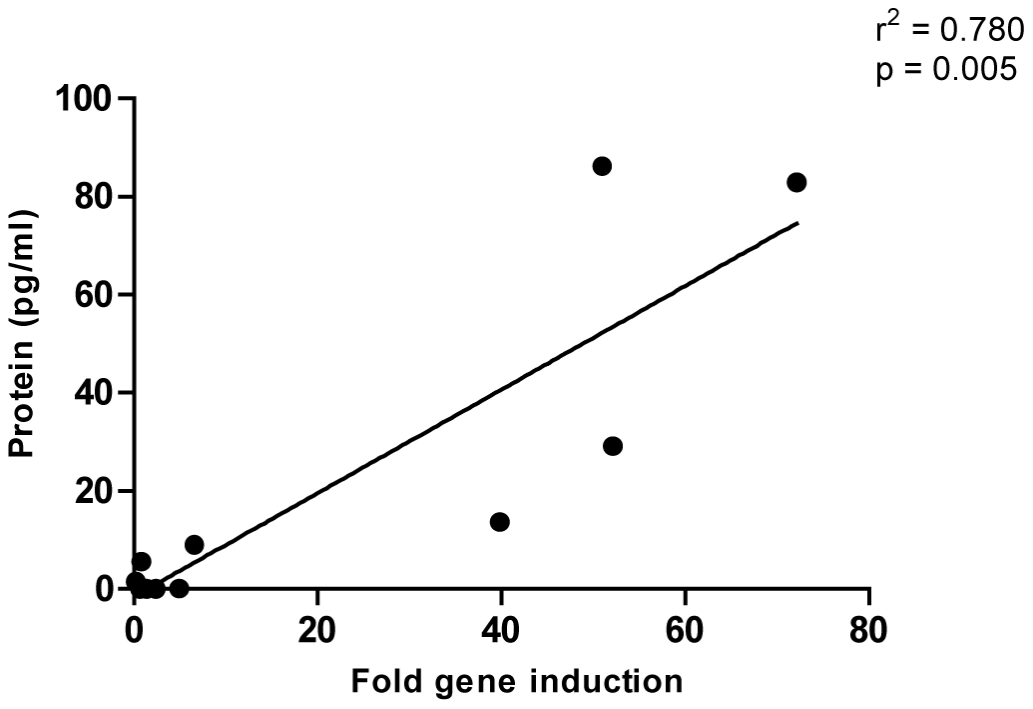
Correlation of SEB induced IFNγ mRNA and protein induction in cervical cells from HIV infected participants. Horizontal lines represent median values. IFNγ mRNA induction was associated to IFNγ protein production in SEB treated samples.

Following stimulation with HIV epitope pool, induction of IL-2 (p = 0.042) but not IFNγ or TNFα (IFNγ, p = 0.138; TNFα, p = 0.144; Wilcoxon Signed Ranks test) was observed compared to medium alone in CMCs. Overall however, median HIV-specific induction in CMCs for all genes remained at 1.0 fold for all cytokines (Figure 3) suggesting that qPCR was unable to detect any HIV-specific immune responses in the genital tract. Nonetheless, robust detection of HIV-specific cellular T cell responses was observed in PBMCs from the same participants (median fold induction; IFNγ, 6.3; TNFα, 2.33; IL-2, 1.3; see Figure 3). CMC incubation with SEB significantly induced mRNA expression of IFNγ above background (median 5.0 fold, p = 0.012), TNFα (median 1.0, p = 0.043) and IL-2 (median 1.9 fold, p = 0.017) as evaluated using the Wilcoxon Signed Ranks test (Figure 4). In addition, SEB-induced cervical IFNγ mRNA expression was strongly associated with supernatant cytokine levels measured by multiplex ELISA (Spearman's rho  = 0.780, p = 0.005; Figure 5) but this was not the case for IL-2 or TNFα levels. In PBMCs, the qPCR platform readily detected SEB-induced cytokine expression above background (IFNγ, 148.8 fold, p = 0.001; IL-2, 130.9 fold, p = 0.008; TNFα, 7.4 fold, p = 0.001; Wilcoxon Signed Ranks test; Figure 4).

After dividing the total cytobrush CMC yield into 3 wells for each treatment, median CMC cell counts after Ficoll separation were 1.93×10^5^ (range 5.33×10^4^−1.33×10^6^ cells) per experiment. There was a positive association between CMC numbers and the induction of IL-2 gene expression by the HIV epitope pool (Spearman's rho  = 0.879; p<0.001) and by SEB (Spearman's rho  = 0.611; p = 0.035). However, no association was observed between CMC numbers and the induction of either IFNγ or TNFα expression by the HIV epitope pool or SEB (all p≥0.1). In addition, input CMC numbers did not correlate with cervical HIV-specific or SEB-induced supernatant cytokine levels by multiplex ELISA (all p>0.4). Therefore, observed IFNγ and TNFα cytokine levels appear to be independent of input cervical cell numbers.

## Discussion

Assay platforms that can reliably quantify HIV-specific cellular immune responses in the genital tract are urgently needed to monitor the immunogenicity of candidate HIV vaccines and elucidate the mucosal immune correlates of HIV transmission and susceptibility. Inherent limitations in sensitivity, ability to measure polyfunctional immune responses and low cervical cell yields often preclude the use of conventional immune assays in this context. Therefore, we designed a qPCR assay with the goal of measuring *ex vivo* HIV-specific cellular T cell responses from cervical cytobrush specimens. The evaluated qPCR assay was robustly able to quantify SEB mediated cytokine induction in both CMCs and PBMCs despite low input cell numbers. However, HIV-specific cellular T cell responses could only be measured in PBMCs.

It is unclear why the qPCR assay was unable to detect HIV-induced cellular T cell responses in cervical cells, particularly in light of the sensitivity demonstrated at low PBMC input numbers. Cervical HIV-specific T cell responses have been found to mirror those present in the blood [8], [9], [10], [26], with a frequency similar to or greater than those in blood [8] and a common ontogeny [26]. While this is not a universal finding [6], it seems unlikely that a true discordance in blood-cervical T cell responses would account for the failure of this QPCR-based technique to detect responses in all 13 participants studied, particularly given the robust T cell responses that were seen in PBMC. Instead, an insufficient sensitivity of the assay to detect responses with this input number of cervical cells seems more plausible. The lack of measurable HIV-specific mRNA immune responses in the genital mucosa may also be due to the lower relative proportion of cervical mononuclear cells that are CD8+ T cells, compared to blood. Less than 5% of cervical mononuclear cells collected via cytobrush sampling are actually T cells [9], while blood CD8+ T cell populations range from 12-23% in HIV infected individuals [27]. The reduced CD8+ T cell proportion may have resulted in diminished qPCR assay sensitivity regardless of high overall cytobrush cervical cell yields. In our study total input cell numbers were counted by trypan blue exclusion on a haemocytometer, and so we were not able to determine the precise proportion of mononuclear cells that were CD8+ T cells in either compartment. This limitation would be better addressed by enumerating T cell subsets via flow cytometry assays, such as the Guava automated cell counter [28]. In addition, the use of invasive or pooled samples from multiple genital sampling techniques, including cervical biopsies, cervicovaginal lavage or the collection of two or more cytobrushes may increase yields of cervical mononuclear cells [8], [29]. Lastly, PCR amplification inhibitors in cervical derived cells have been reported elsewhere [30], [31], [32], and this may also have diminished the ability to detect cellular immune responses in the genital tract via qPCR. However, the identification and impact of mucosal qPCR inhibitors on measuring cervical cellular immune responses were not evaluated here and require further investigation to ascertain their impact.

Despite the lack of HIV-specific immune responses, SEB induced responses in CMCs were detected by qPCR in most (but not all) participants, indicating that the assay was functional in cervical cells. SEB cross-links T cell receptors (TCRs) and MHC complexes activating both CD4 and CD8 T cells regardless of antigen specificity [33], unlike the 8-mer HIV epitope pool that only stimulates HIV-specific CD8+ T cells [34]. The broader immune activation profile afforded by SEB may therefore facilitate enhanced detection of mRNA induction at the cervical mucosa. In addition, the higher affinity constant for SEB-TCR binding and the mechanism of SEB cross-linking and stabilization of the TCR-MHC during activation may result in higher avidity for SEB superantigen binding compared to individual peptide-mediated MHC-TCR interactions [35]. Consequently, greater T cell activation may have induced higher cytokine mRNA levels following stimulation with SEB superantigen relative to HIV epitope pool stimulation allowing for mRNA detection. However, the evaluation of superantigen and antigen specific interactions with TCR is beyond the scope of this report. In a minority of participants, cervical SEB-induced immune responses were not always detectable by qPCR. A lack of viable cervical cells would be one possible explanation, and cell viability was not ascertained in these experiments. However, SEB-treated cervical cells from both HIV-infected and uninfected women may sometimes be unresponsive in flow cytometry-based assays despite the demonstration of cell viability via a live/dead gating strategy (unpublished; McKinnon L and Kaul R), and so a lack of cell viability is unlikely to explain these results.

The expression of target gene must be corrected to account for variation in the quantity and quality of extracted RNA, reverse transcription and PCR amplification efficiencies and to measure the relative quantities of target genes[36]. However, the expression of housekeeping genes also varies under different experimental conditions [24], [36], [37]. Therefore we screened a panel of five commonly used housekeeping genes in CMC and PBMC to evaluate their stability under different treatment conditions, and found GAPDH and β-actin genes to be the most stable housekeeping genes, respectively. Our findings agree with one [25] but not another [24] of only two prior studies evaluating qPCR to measure gene expression *ex vivo* in cervical tissue. However, these studies were not using qPCR to assay antigen-specific responses where short-term stimulation might alter the stability of potential housekeeping genes. For this reason we used the NormFinder software to directly evaluate intergroup stability of candidate housekeeping genes in our immunostimulated samples[37]. In addition, our selected housekeeping genes may have varied from other reports as our samples were derived from HIV infected participants. HIV has been demonstrated to exert substantial effects on the transcription pathways of several classes of genes [38], including commonly used “housekeeping genes” selected in the above mentioned studies [39]. This study therefore highlights the importance of selecting a stable reference gene for normalization of target gene expression based on empirical evaluation for a given assay condition, cell type and infection status.

No IL-2 protein was detected in cell culture supernatants using the cytometric bead array, despite the detection of low-level IL-2 mRNA levels by qPCR. In other studies describing discordance between mRNA detection and protein synthesis it has been postulated that a few highly transcriptionally active cells may permit detection of cytokine mRNA, but that these highly active cells do not secrete enough protein to be quantified using protein assays[40]; in this scenario IL-2 protein would be secreted into the cell supernatant but fall below the lower limit of detection of the CBA assay (20 pg/ml). Discordant kinetics between mRNA induction and protein production is another possible explanation. Finally, since our culture experiments did not block the IL-2-receptor, any IL-2 produced may have been consumed by activated immune cells [23], [41].

In summary, we describe the process of development and testing of a real-time quantitative PCR assay to measure *ex vivo* cervical HIV-specific cellular T cell responses. We demonstrate an appropriate algorithm for the selection of housekeeping genes, and determine the optimal cytokine induction kinetics and cell input numbers for measuring such responses in the blood. However, the resulting optimized qPCR assay was not able to detect HIV-specific cellular T cell responses in the cervix of HIV-infected women despite similar input cell numbers, and despite the demonstration of a robust immune response in the PBMCs from the same participant. The reasons for this result are not clear, but we believe that the description of the assay development process will be of use to those in the mucosal vaccine field who are endeavouring to develop robust platforms to evaluate vaccine-induced T cell responses in the female genital tract.

## Methods

### Ethics statement

All participants provided written informed consent, and the study protocol was reviewed and approved by the Research Ethics Boards at the University of Nairobi and the University of Toronto.

### Sample processing and in vitro antigenic stimulation

Blood samples and cervical cytobrush specimens were obtained from HIV infected participants in Canada and Kenya who were not actively menstruating, under research protocols approved by the Universities of Toronto, Manitoba, and Nairobi as well as The Kenyatta National Hospital. All HIV-infected participants had a blood CD4+ T cell count above 350/mm^3^, and were antiretroviral therapy naïve. Sample collection and processing was performed as previously described [42]. Briefly, cervical samples were obtained by first scraping the external cervical os using a plastic cell scraper (Benzi Jinshuo Applicator Co) and a cervical histobrush (Histobrush Spectrum Lab) was then inserted into the cervical os and rotated through 360°. A mini-cervical lavage was then performed using 1 ml PBS to collect loosened cells. All genital tract samples were then combined into a 50 ml sample collection tube containing 10 mls of PBS and transported to the laboratory on ice within 2 hours. Blood was collected by venipuncture into heparin Vacutainer tubes (BD Bioscience). Peripheral blood mononuclear cells (PBMC) and cervical mononuclear cells (CMCs) were isolated by density gradient centrifugation. Samples were washed twice and resuspended in RPMI 1640 media with 10% heat inactivated fetal bovine serum, 1% Penicillin and 1% streptomycin (R10; FBS; Sigma). PBMCs were then used fresh for stimulation experiments or cryopreserved at −150°C in FBS containing 10% DMSO until use. All cervical T cell assays were performed using freshly obtained cytobrush samples. Both PBMC and CMC numbers were determined by light microscopy, based on trypan blue exclusion.

PBMC and CMC samples were incubated with; i) a pool of 49 immunodominant CD8+ optimized HIV epitopes (HIV epitope pool) restricted by a range of class I HLA alleles, at a concentration of 2.0 ug/ml/peptide; ii) R10 tissue culture medium alone or iii) staphylococcal enterotoxin B superantigen (SEB; Sigma) at 3.0 ug/ml. The use of predefined, immunodominant CD8+ HIV epitopes from multiple HIV clades and restricted by a variety of different HLA types would allow for measurement of CD8+ immune responses in most participants. Kinetics and sensitivity experiments utilized a variety of input cell numbers and incubation durations (see results) at 37°C in 5% CO_2_. Cell pellets and supernatants were harvested by centrifugation at 10,000 rpm for 5 minutes and placed in RNA later solution (Ambion). Culture supernatants were stored as is at −80°C prior to qPCR or protein measurements.

### RNA extraction

RNA was extracted from cell pellets and genomic DNA was eliminated using the Qiagen RNAeasy Plus Kit (Qiagen) as per manufacturer's instructions. RNA was eluted in 30 ul of nuclease free water. The quantity of extracted RNA was evaluated using the Nanodrop ND1000 (Thermofisher Scientific). To ensure the integrity of RNA, only samples with a 260/280 OD ratio of between 1.7–2.0 were used.

### Reverse Transcription and qPCR

Complementary DNA (cDNA) was created by adding 10 ng of RNA to the reverse transcription master mix and reverse-transcribed using the Superscript III Kit (Invitrogen) as per manufacturer's instructions. The cDNA product was then diluted 1∶7 in nuclease free water and the samples stored at −20°C. Genomic DNA (gDNA) standards were isolated from purified placental tissue and used as a universal standard over a 7-log (36 ng – 0.4 ng) dilution range to calculate relative gene expression levels [43]. A broad dynamic range of standards was used to account for the varied expression levels of different targeted genes. Single intra-exon gene-specific primers were generated using Primer Express Software (Perkin Elmer Applied Biosystems). Each primer pair was evaluated for specificity by melting curve analysis to ascertain that only one product was amplified and a BLAST search was performed to confirm that primer sequences amplified only the target gene of interest. Primer pairs generating multiple peaks (indicative of primer-dimer artifacts or non-specific gene amplification), or primers with less than 90% primer-pair amplification efficiency were discarded. The final primer pairs used are listed in Table 2.

**Table 2:** Primer sequences for SYBR green Real-Time PCR.

mRNA Target	Forward Primer Sequences 5′→3′	Reverse Primer Sequences 3′→5′
**IFNγ**	AGGGAAGCGAAAAAGGAGTCA	GGACAACCATTACTGGGATGCT
**TNFα**	GCCAGAATGCTGCAGGACTT	GGCCTAAGGTCCACTTGTGTCA
**IL2**	ATGAGACAGCAACCATTGTAGAATTT	CACTTAATTATCAAGTCAGTGTTGAGATGA
**β-Actin**	TCCCTTGCCATCCTAAAAGCCACCC	CTGGGCCATTCTCCTTAGAGAGAAG
**TBP**	GGGCATTATTTGTGCACTGAGA	TAGCAGCACGGTATGAGCAACT
**GAPDH**	TGGACCTGACCTGCCGTCTA	CCCTGTTGCTGTAGCCAAATTC
**B2M**	GAGTGCTGTCTCCATGTTTGATGT	AAGTTGCCAGCCCTCCTAGAG
**HPRT**	GCCTATAGACTATCAGTTCCCTTTGG	TGCTGTGGTTTAAGAGAATTTTTTCA

Interferon Gamma (IFNγ); Tumour Necrosis Factor alpha (TNFα); Interleukin 2 (IL2); β-2-Microglobulin (B2M); TATA box binding protein (TBP); Glyceraldehyde-3-phosphate dehydrogenase (GAPDH); Ribosomal Protein L32 (RPL32); human hypoxanthine phosphoribosyltransferase (HPRT).

The qPCR assay was performed in a 10 ul volume in a 384-well plate. ABI Prism 7900HT (Applied Biosystems) and SYBR green fluorescent dye was used to detect amplification under the following amplification conditions: i) 1 warm-up cycle for 2 min at 50°C ii) 1 pre-amplification cycle for 10 minutes at 95°C, 40 amplification cycles for 15 seconds at 95°C and for 1 minute at 60°C, iii) end-amplification cycle for 15 seconds at 95°C, 15 seconds at 60°C and 15 seconds at 95°C. All reactions were run in triplicate with a non-template control blank) for each primer pair to control for contamination or primer-dimers. Quantitative PCR values crossing threshold (Ct) were obtained during the exponential amplification phase using SDS 2.3 Software (Applied Biosystems) analysed for respective gene quantities and exported into Microsoft Excel for further analysis.

### Cytokine Protein Quantification

Cytokine concentrations (pg/ml) from culture supernatants were determined using the flow cytometry based Cytokine Bead Array Kit (CBA; BD Biosciences) or the Searchlight Multiplex-ELISA (M-ELISA; Aushon Biosystems) as per manufacturer's instructions. Interferon Gamma (IFNγ), Tumour Necrosis Factor alpha (TNFα) and Interleukin 2 (IL-2) cytokines were assayed. The CBA limit of detection for IFNγ, TNFα, IL-2 was 20 pg/ml for all evaluated analytes while the M-ELISA limit of detection for IFNγ, TNFα, and IL-2 was 0.5, 0.5, 0.1 pg/ml respectively. CBA samples were run in unicate while M-ELISA samples were assayed in duplicate. Background (basal) cytokine levels were subtracted from presented antigen and superantigen specific cytokine levels.

### Statistical analysis

Gene quantities were calculated from standard curves in arbitrary units and values were then analysed using the NormFinder model within a Microsoft Excel add-on (http://mdl.dk/publicationsnormfinder.htm). The most stable housekeeping gene was selected to normalize target gene expression levels as described elsewhere [19]. Briefly, to assess antigen-specific gene induction, results for each target gene were normalized by dividing the amount of the amplified gene target by the amount of the housekeeping gene for each sample. This ratio was then further divided by the respective negative control (medium alone) at the given time point, and reported as a Fold gene-induction ratio [19]. Non-parametric Mann-Whitney U and Wilcoxon Signed Ranks statistical analyses were performed using SPSS Version 17.0 software (SPSS Inc.) to analyse the relationship between gene and protein expression over different treatments.
